# SOT101 induces NK cell cytotoxicity and potentiates antibody-dependent cell cytotoxicity and anti-tumor activity

**DOI:** 10.3389/fimmu.2022.989895

**Published:** 2022-10-10

**Authors:** Zuzana Antosova, Nada Podzimkova, Jakub Tomala, Katerina Augustynkova, Katerina Sajnerova, Eva Nedvedova, Milada Sirova, Guy de Martynoff, David Bechard, Ulrich Moebius, Marek Kovar, Radek Spisek, Irena Adkins

**Affiliations:** ^1^ Preclinical Department, SOTIO Biotech a.s, Prague, Czechia; ^2^ Department of Immunology, 2nd Faculty of Medicine and University Hospital Motol, Charles University, Prague, Czechia; ^3^ Laboratory of Tumor Immunology, Institute of Microbiology of the Czech Academy of Sciences, Prague, Czechia; ^4^ Cytune Pharma, Nantes, France

**Keywords:** NK cells, antibody-dependent cytotoxicity, interleukin-15, immunotherapy, therapeutic antibodies, RLI-15

## Abstract

SOT101 is a superagonist fusion protein of interleukin (IL)-15 and the IL-15 receptor α (IL-15Rα) sushi+ domain, representing a promising clinical candidate for the treatment of cancer. SOT101 among other immune cells specifically stimulates natural killer (NK) cells and memory CD8^+^ T cells with no significant expansion or activation of the regulatory T cell compartment. In this study, we showed that SOT101 induced expression of cytotoxic receptors NKp30, DNAM-1 and NKG2D on human NK cells. SOT101 stimulated dose-dependent proliferation and the relative expansion of both major subsets of human NK cells, CD56^bright^CD16^-^ and CD56^dim^CD16^+^, and these displayed an enhanced cytotoxicity *in vitro*. Using human PBMCs and isolated NK cells, we showed that SOT101 added concomitantly or used for immune cell pre-stimulation potentiated clinically approved monoclonal antibodies Cetuximab, Daratumumab and Obinutuzumab in killing of tumor cells *in vitro*. The anti-tumor efficacy of SOT101 in combination with Daratumumab was assessed in a solid multiple myeloma xenograft in CB17 SCID mouse model testing several combination schedules of administration in the early and late therapeutic setting of established tumors *in vivo*. SOT101 and Daratumumab monotherapies decreased with various efficacy tumor growth *in vivo* in dependence on the advancement of the tumor development. The combination of both drugs showed the strongest anti-tumor efficacy. Specifically, the sequencing of both drugs did not matter in the early therapeutic setting where a complete tumor regression was observed in all animals. In the late therapeutic treatment of established tumors Daratumumab followed by SOT101 administration or a concomitant administration of both drugs showed a significant anti-tumor efficacy over the respective monotherapies. These results suggest that SOT101 might significantly augment the anti-tumor activity of therapeutic antibodies by increasing NK cell-mediated activity in patients. These results support the evaluation of SOT101 in combination with Daratumumab in clinical studies and present a rationale for an optimal clinical dosing schedule selection.

## Introduction

IL-15-based immunotherapy has gained a particular attention in recent years (1) due to a better safety profile in comparison to the high dose IL-2 therapy which was approved for treatment of malignant melanoma and renal cell carcinoma (2). Despite sharing similar properties with IL-2 (3) such as activation of NK, NKT, γδ T cells and cytotoxic CD8^+^ T lymphocytes, IL-15 bears advantages over IL-2 in stimulation and maintenance memory CD8^+^ T cell response (4), lack of activation-induced cell death (5), T regulatory cell expansion or inducing capillary leak syndrome (6).

SOT101 (also known as RLI-15 or SO-C101) is a fusion protein that consists of the N-terminal sushi+ domain of the human IL-15Rα covalently coupled *via* a non-immunogenic linker of Glycine-Serine residues to the mature IL-15 sequence (7, 8). SOT101 acts as a selective and potent agonist of the IL-15 pathway through the IL-2/IL-15Rβγ thereby inducing proliferation and activation of CD8^+^ T cells, memory CD8^+^ T cells, NK cells, γδ T cells and NKT cells *in vitro* and *in vivo* without the expansion of T regulatory cells. SOT101 was shown to promote NK cell development and differentiation in human immune system (HIS) mice (9), to stimulate T cell reconstitution and T cell-dependent antibody responses in HIS mice (10). SOT101 mediated a stronger anti-metastatic activity and anti-tumor efficacy in various mouse cancer models over IL-15 (11). SOT101 was shown to synergize with an anti-PD-1 antibody in mouse colorectal cancer models (12). Finally, SOT101 has been investigated in a dose escalation Phase I clinical trial as monotherapy, or in combination with pembrolizumab for treatment of patients with advanced solid tumors (NCT04234113) and is currently under efficacy and safety evaluation in Phase II clinical trial in combination with pembrolizumab in patients with selected advanced solid tumors (NCT05256381). In addition, SOT101 was shown to synergize with the anti-GD2 or anti-CD20 antibodies when targeted in the form of an immunocytokine to the tumor in preclinical mouse tumor models (13, 14) mainly *via* increasing NK cell activity. However, SOT101 potential to increase the benefit of antibody-dependent cell cytotoxicity (ADCC)-inducing therapeutic antibodies used in the clinical praxis to treat selected malignant diseases and the potential optimal scheduling of these drugs with relevance to dosing in patients has not been investigated.

Natural killer (NK) cells are considered a part of type I innate-like cells whose survival and development depends highly on cytokines, predominantly IL-2 and IL-15 (15, 16). Beside their role in a defense against viral infection, NK cells play an important role in the control of the tumor development and progression (17). NK cells can kill primary tumor cells, inhibit metastatic migration and colonization to distant tissues (18). By producing many cytokines such as IFN-γ and chemokines and due to their interaction with dendritic cells (19) NK cells also shape adaptive immune responses (20, 21). Cytotoxic action of NK cells is driven by the “missing self” and “induced self” modes which are determined by the interplay among the activating co-stimulatory and inhibitory signals represented by various types of functional receptors (22, 23). The integration signals then determine the NK cell function (17, 22). In humans, NK cells are defined as CD3^-^CD56^+^ (24) and can be broadly divided into two main subpopulations CD56^dim^ and CD56^bright^ based on the expression of the CD56 marker. These NK cell subtypes further divide based on their primary function and site of location into circulating or tissue resident NK cells (17). In peripheral blood circulating CD56^bright^ cells represent only 10% of NK cells and home predominantly to secondary lymphoid tissues (25). These cells express very little or no CD16 receptor and are considered as immunoregulatory NK cells producing proinflammatory cytokines, also upon activation by IL-15 (26, 27). Although considered mainly as producers of cytokines, it has been shown that CD56^bright^ NK cells are able to perform cytotoxic function, specifically after activation by IL-15, against tumor cells *in vitro* and *in vivo* (28, 29). CD56^dim^ phenotype of NK cells represent the most of circulating NK cells (90%) found in peripheral blood. These cytotoxic NK cells exert rapid action *via* release of a high number of granules containing cytolytic enzymes such as perforin and granzymes and express CD16 (FcγRIIIa) receptors (30) which are important for antibody-dependent cellular cytotoxicity (ADCC). ADCC is a mechanism where the Fc part of an antibody bound to its target, e.g., a tumor cell engages the CD16 receptor on the NK cell surface which then upon receptor crosslinking leads to degranulation of NK cells and lysis of the target tumor cells. ADCC is a specifically important mechanism for the action of monoclonal therapeutic IgG1 antibodies used in the clinics used for treatment of many cancer indications (31, 32). Besides ADCC, therapeutic IgG1 antibodies exert their function also *via* direct cell death inducing properties, complement-dependent cytotoxicity, or antibody-dependent phagocytosis (33). The major success of the therapeutic antibodies is documented by many approved compounds in the clinics among others Daratumumab, Cetuximab or ADCC-enhanced Obinutuzumab (31). IL-15 was shown to potentiate ADCC of some of these antibodies *in vivo* and *in vitro* (13, 14, 34–36).

In this study, we investigated the expression of activation receptors and the cytotoxic activity of SOT101-treated NK cells and their subpopulations towards tumor cells *in vitro*. We evaluated the capacity of SOT101 to potentiate ADCC-mediated tumor cell killing induced by Daratumumab, Cetuximab and Obinutuzumab *in vitro*. Most importantly, we investigated whether SOT101 in combination with Daratumumab can additively increase anti-tumor efficacy in a human multiple myeloma xenograft tumor model *in vivo* and we identified the optimal schedules of the drug sequencing in the early and late therapeutic setting of established tumors. These data suggest that SOT101 can be combined with therapeutic antibodies in patients. Specifically, the sequential combination of Daratumumab followed by SOT101 administration or a concomitant administration of both drugs, might achieve the best anti-tumor efficacy.

## Material and methods

### SOT101

SOT101 also known as RLI-15 or SO-C101 (CAS number 1416390-27-6) is a proprietary compound of Sotio Biotech AG, Switzerland. SOT101 is a fusion protein that consists of the NH2-terminal (amino acids 1–77, sushi+) domain of IL-15Rα coupled *via* a 20–amino acid linker to IL-15 (7, 8). SOT101 was expressed in CHO-S cells and purified to ≥ 95% purity.

### Human cancer cell lines *in vitro*

The Burkitt’s Lymphoma cell line Daudi (CCL-213™) expressing CD38 and CD20 and head and neck squamous cell carcinoma SCC25 (CRL-1628™) cell line expressing EGFR were obtained from the American Type Culture Collection (ATCC, USA). Daudi cells were cultivated in RPMI 1640 medium (ATCC modification) (Gibco, Thermo Fisher Scientific, USA) supplemented with 10% heat-inactivated fetal bovine serum (Sigma Aldrich, USA) and 100 U/ml penicillin + 100 mg/ml streptomycin (Gibco, Thermo Fisher Scientific, USA), at 37°C in a humidified atmosphere containing 5% CO_2._ SCC25 cells were cultivated in a 1:1 mixture of Dulbecco’s modified Eagle’s medium (DMEM) and Ham’s F12 medium (Gibco, Thermo Fisher Scientific, USA) supplemented with 400 ng/ml hydrocortisone (Sigma Aldrich, USA), 10% heat-inactivated fetal bovine serum and 100 U/ml penicillin + 100 mg/ml streptomycin at 37°C in a humidified atmosphere containing 5% CO_2_. K562 (CCL-243™) CML lymphoblast cell line was obtained from ATCC. K562 were cultivated in DMEM supplemented with 10% heat-inactivated fetal bovine serum at 37°C in a humidified atmosphere containing 5% CO_2_. The K562 cell line is widely used as a highly sensitive *in vitro* target for the natural killer assay.

### Phenotypic analyses and proliferation of SOT101-stimulated human NK cells

Peripheral blood mononuclear cells (PBMC) were obtained from buffy coats of healthy donors (n=8) by Ficoll-Paque™ PLUS Media gradient centrifugation (Thermo Fisher Scientific, USA). PBMC were cultivated in RPMI 1640 medium supplemented with 2 mM GlutaMAX I CTS, 100 U/ml penicillin + 100 mg/ml streptomycin, 1 mM Sodium pyruvate, 1% non-essential amino acids, 50 μM 2-Mercaptoethanol (all from Gibco, Thermo Fisher Scientific, USA) and 10% AB human serum (heat-inactivated) (Invitrogen, USA). 1 × 10^6^/ml PBMC were incubated with SOT101 at concentrations of 0, 0.001, 0.01, 0.1, 1 and 10 nM for 7 days at 37°C in a humidified atmosphere containing 5% CO_2_. The phenotype of NK cell populations and their relative expansion and proliferation was determined by flow cytometry on day 3 and 7 using following fluorescently labeled antibodies: CD3-APC-eFluor780, CD4-eFluor450, Ki67-APC (all three from eBioscience, USA), CD8-PE-DlyLight594 (Exbio, Czech Republic), CD56-Aexa Fluor700, CD16-PE-Cy7 (both from Biolegend, USA). The dead cells detected by LIVE/DEAD Fixable Aqua stain (Thermo Fisher Scientific, USA) were excluded from the analyses. The gating strategy was as follows: singlets/live lymphocytes/CD3^-^/CD56 vs. CD16 or CD56^+^Ki67^+^.

### Flow cytometry staining

For flow cytometry staining, PBMC or suspension of mouse splenocytes were placed into a V-shape 96 well plate, centrifuged 2200 rpm for 2 min and supernatant was discarded. Cells were stained with a mixture of the appropriate extracellular antibodies and LIVE/DEAD Fixable stain (Thermo Fisher Scientific, USA) in FACS buffer (PBS from Lonza, Switzerland supplemented with 0.2% BSA from Sigma Aldrich, USA) (50 µl/sample/well) for 30 min at 4°C. After a washing step with FACS buffer, cells were fixed with 100 µl/well fixation buffer (1 fixation concentrate: 3 fixation diluent) (eBioscience, USA) for 20 min at 4°C. Then 100 µl/well of 1x permeabilization buffer (diluted from 10x with dH2O) (eBioscience, USA) was added and cells were centrifuged 2200 rpm, 2 min. Supernatant was discarded and 100 µl/well of permeabilization buffer (eBioscience, USA) was added. Cells were centrifuged 2200 rpm, 2 min, supernatant discarded and stained with intracellular antibody (Ki67 or IFN-γ detection antibodies) (50 µl/sample) in PBS containing 2 ul of rat serum/50 µl (Sigma Aldrich, USA). Cells were incubated for 20 min at 4°C. After a washing step, 120 µl/well of FACS buffer was added to the cells for measurement. Data were collected using Flow cytometer LSR Fortessa (Becton Dickinson, USA) and Software BD DiVA. FlowJo (Tree Star, Inc., USA) software was used for the cytometric data evaluation.

### Detection of the expression of cytotoxic receptors on human NK cells

PBMC were obtained from buffy coats of healthy donors (n=4) by Ficoll-Paque™ PLUS Media gradient centrifugation (Thermo Fisher Scientific, USA). PBMC were treated as described above and the expression of activating and inhibitory receptors on NK cells was determined by flow cytometry on day 3 and 7 using following fluorescently labeled antibodies in two panels: CD45-PE-Dy594 (Exbio, Czech Republic), CD3-APC-eFluor780 (eBioscience, USA), NKG2D-PE-Cy7, CD56-Alexa Fluor700, CD16-PE-Cy7, DNAM-1-FITC (all from Biolegend, USA), NKp30-PE, NKG2A-APC (R&D), CD158a-PE and CD158b-FITC (BD Biosciences, USA). The dead cells detected by LIVE/DEAD Fixable Aqua stain (Thermo Fisher Scientific, USA) were excluded from the analyses. The gating strategy was as follows: singlets/live CD45^+^/CD3^-^/CD56^+^/receptor^+^. Mean fluorescence intensity (MFI) of receptor expression on NK cells was determined from CD3^-^CD56^+^ cell population.

### Human NK cell cytotoxicity and tumor cell killing assay

The SOT101-stimulated cytotoxic activity of human NK cells and their subsets towards the target K562 cells was analyzed by flow cytometry using LAMP-1 staining as a marker of cytotoxic degranulation, intracellular IFN-γ staining and detection of dead tumor cells (DAPI^+^). PBMCs (n=4) were pre-stimulated with 0, 0.1, 1 and 10 nM SOT101 for 3 days, then added to the target tumor cells K562 (4 × 10^4^) at E:T ratio 1:5 or left in the well without K562 to determine the spontaneous cytotoxic activity for 4 h at 37°C in a humidified atmosphere containing 5% CO_2_. LAMP-1-APC antibody was added (4 µl/well) at the beginning of cell coincubation. The killed K562 cells were detected after 6 h at 37°C in a humidified atmosphere containing 5% CO_2_. K562 had been fluorescently labeled by DiD (Thermo Fisher Scientific, USA) (25 µl/5 × 10^6^ K562 cells) in RPMI 1640 medium without serum and supplements for 15 min at 37°C before the cocultivation experiment *in vitro* was conducted. Following fluorescently labeled antibodies were used: CD45-PE-DyLight594, CD16-Alexa Fluor700 (Exbio, Czech Republic), CD3-eFluor780, CD8-eFluor450, IFN-γ-PE-Cy7 (eBioscience, USA), CD56-PE, LAMP-1-APC (Biolegend, USA). The dead cells detected by LIVE/DEAD Fixable Aqua stain (Thermo Fisher Scientific, USA) were excluded from the analyses. In survival experiments, the percentage of dead K562 tumor cells was determined by DAPI (both from Thermo Fisher Scientific, USA) staining and gating on DiD^+^ K562 cells. The gating strategy was as follows: singlets/live CD45^+^/CD3^-^/CD56^+^/LAMP-1 vs. IFN-γ or singlets/CD45^-^/DiD^+^DAPI^+^.

### Human PBMC cytotoxicity assay with SOT101 and monoclonal antibodies

PBMC (n= 3) were cultivated in RPMI 1640 medium supplemented with 2 mM GlutaMAX I CTS, 100 U/ml penicillin + 100 mg/ml streptomycin, 1 mM Sodium pyruvate, 1% non-essential amino acids, 50 μM 2-Mercaptoethanol (all from Gibco, Thermo Fisher Scientific, USA) and 10% AB human serum (heat-inactivated) (Invitrogen, USA). In concomitant cytotoxicity experiments, PBMCs were added to the target tumor cells together with 1 nM SOT101 and the antibodies at concentrations of 0.1, 1 and 10 nM, and incubated for 20 h at 37°C in a humidified atmosphere containing 5% CO_2._ E:T ratio was 25:1 for Daudi cells incubated with Daratumumab (Darzalex™, Janssen Biotech Inc., USA) and Obinutuzumab (Gazyvaro, Roche, Switzerland) E:T ratio was 150:1 for SCC25 incubated with Cetuximab (Erbitux, Eli Lilly, USA and Company/Merck KGaA, Germany). In sequential cytotoxicity experiments, PBMCs were pre-stimulated with 0.1 nM SOT101 for 3 days (SOT101-PBMC) and then added to the target tumor cells together with antibodies at 0.1, 1 and 10 nM and incubated for 24 h at 37°C in a humidified atmosphere containing 5% CO_2_. E:T ratio was 15:1 for Daudi cells and 100:1 for SCC25 cells, respectively. Adherent SCC25 cells were seeded in the tissue culture plate 24 h before the addition of PBMC at 37°C in a humidified atmosphere containing 5% CO_2_. 1 × 10^4^ Daudi cells per well and 2 × 10^3^ SCC25 cells were used for the experiment. Cytotoxicity was determined by a Cytotoxicity Detection Kit^PLUS^ Lactate dehydrogenase release assay (LDH, Thermo Fisher Scientific, USA) according to the manufacturer’s instructions. The cytotoxicity was determined according to the following formula where high control was represented by Daudi cells incubated with a lysis solution and low control included non-treated Daudi cells.


$$Cytotoxicity\;(\%)=\;\frac{sample-NK\;only-Low\;control}{High\;control-Low\;control}\;\times 100$$

In some experiments, the direct tumor cell killing and LAMP-1 as a NK cell degranulation marker of cytotoxicity were detected by flow cytometry. PBMCs were pre-stimulated with 0.1 nM SOT101 for 3 days (SOT101-PBMC) and then added to the target tumor cells at E:T ratio was 1:1 together with Daratumumab at 0.1, 1 and 10 nM and incubated for 4 h at 37°C in a humidified atmosphere containing 5% CO_2_. Daudi cells had been fluorescently labeled by DiD (Thermo Fisher Scientific, USA) (25 µl/5 × 10^6^ Daudi cells) in RPMI 1640 medium without serum and supplements for 15 min at 37°C before the cocultivation experiment *in vitro* was conducted. LAMP-1 antibody was added to the assay at the beginning of cell incubation. The following fluorescently labeled antibodies were used to determine the NK cell cytotoxic activity by flow cytometry: CD45-BV605 (HI30 clone) (BD Biosciences, USA), CD3-APCeFluor780 (OKT3 clone) (Thermo Fisher Scientific, USA), CD56-FITC (MEM-188 clone), CD8-PE-Dylight (MEM-31), CD16-PECy7 (Biolegend, USA) and LAMP-1 (CD107a)-PE (H4A3 clone) (Exbio, Czech Republic). The gating strategy was as follows: singlets/live DAPI^-^CD45^+^/CD3^-^/CD56^+^ vs LAMP-1^+^. The dead tumor cells were detected as DiD^+^DAPI^+^.

### Human NK cell isolation and killing assay with Daratumumab

Human NK cells were isolated from PBMC (n=4) of healthy donor buffy coats using NK cell isolation kit (Miltenyi Biotec, Germany) according to the manufacturer’s instructions. Isolated human NK cells were treated with SOT101 (0.1 nM, 2 days) and then incubated with Daratumumab (Darzalex™, Janssen Biotech Inc., USA) and Daudi tumor cells (E:T 10:1) for 4 h. Cell cytotoxicity was determined by a lactate dehydrogenase release assay (LDH, Thermo Fisher Scientific, USA) as stated above using appropriate controls.

### Detection of *in vivo* SOT101-stimulated mouse NK cells

Pharmacodynamic activity of SOT101 at various doses, route of administrations and schedules was investigated in healthy C57BL/6 mouse (11-12 weeks of age). Mice were obtained from the breeding colony at the Institute of Microbiology of the Czech Academy of Sciences. All animal experiments were approved by the Animal Welfare Committee of the Institute of Microbiology of the Czech Academy of Sciences, in accordance with the Guidelines for the Care and Use of Laboratory Animals, the Act of Czech National Assembly, the Collection of Laws no. 246/1992. Permissions no. 110/2016 were issued by the Animal Welfare Committee of the Institute of Microbiology of the Czech Academy of Sciences in Prague. SOT101 was diluted and administered in 0.9% NaCl (Sigma Aldrich, USA) or PBS (Lonza, Switzerland) as one daily injection intraperitoneally (IP) or subcutaneously (SC) at day 1 and based on the schedule tested on the consecutive or non-consecutive days always at least 24h apart Analysis of NK cells from spleens and vascular leak syndrome (VLS) as determined by the lung wet weight was performed on day 5 from 2-4 mice in each group. Spleens were transferred into the gentleMACS C Tubes containing 5 ml FACS buffer with 2 mM EDTA (Sigma Aldrich, USA). Single cell suspension was obtained using the GentleMACS dissociator (Miltenyi Biotec, Germany) according to the manufacturer’s protocol. Cell suspension was passed through the 70 µm strainer (CellTrics, Sysmex, Japan) into the 50 ml falcon tubes and centrifuges at 1200 rpm for 10 min. at 4°C. Red blood cells were lysed by addition of 1 ml of ACK lysing buffer (Gibco, USA) for 10 min. Then 1ml of FACS buffer with 2 mM EDTA was added, passed through the 30 µm strainer (CellTrics, Sysmex, Japan) and centrifuged at 1200 rpm for 10 min. at 4°C. The phenotype of NK cell population and the relative expansion and proliferation were determined by flow cytometry using following fluorescently labeled Bioscience antibodies: CD3-PE-Cy7, DX5-eFluor450, Ki67-APC (all three from eBioscience, USA), CD8-V500, CD4-PerCP (both BD Biosciences, USA). The dead cells detected by LIVE/DEAD Fixable Dye780 stain (eBioscience, USA) were excluded from the analyses. The gating strategy was as follows: singlets/live lymphocytes/CD3^-^/DX5^+^/Ki67. Data were collected using Flow cytometer BD LSR II (Becton Dickinson, USA) and Software BD DiVA. FlowJo (Tree Star, Inc., USA) software was used for the cytometric data evaluation. To determine the vascular leakage syndrome (VLS) in mice after the SOT101 treatment, lungs were gently taken out from mouse, placed into a microcentrifuge tube and the lung weight was determined. Then lungs were lyophilized for 10 h. The net weight difference between the wet and dry lungs was used to determine VLS.

### Pharmacokinetics of SOT101 in mice

SOT101 at 2.5 mg/kg was administered subcutaneously (SC) as a single dose diluted in 0.9% NaCl (Sigma Aldrich, USA) in C57BL/6 mice (two mice per one time point). Serum samples were collected at 2 min, 15 min, 1 h, 2 h, 4 h, 6 h, 8 h, 24 h and 32 h after dose administration and stored at -20°C until analysis by ELISA assays. ELISA plate was coated with 100 µl/well of coating solution consisting of 1 μg/ml rabbit anti-RLI-15-PR01 antiserum (Agro-Bio, France) in DPBS (Sigma Aldrich, USA) and incubated at 4°C for 14 h. After every incubation step plate was washed four times with 200 µl/well of wash buffer (PBS-Tween20). 200 µl/well of blocking buffer (Thermo Fisher Scientific, USA) was added and incubated at room temperature (RT) for 60 min. After a washing step, 100 µl/well of standard, calibration and QC samples prepared in in Low Cross Buffer (Candor Bioscience, Germany) with 10% mouse serum (Sigma Aldrich, USA) were added and incubated RT for 90 min (± 5 min) with agitation. Plates were washed and 100 µl/well of detection solution consisting of 0.5 µg/ml human/primate anti-IL-15 biotinylated antibody BAM247 (R&D systems, USA) in Low Cross Buffer was added and incubated at RT for 90 min (± 5 min) with agitation. Plates were washed, 100 µl/well of streptavidin-HRP (BD Biosciences, USA) solution was added and incubated RT for 30 min (± 5 min) with agitation. After a washing step, 100 µl/well of TMB Substrate (Sigma Aldrich, USA) was added and incubated at RT until the samples turned blue for max. 15 min (usually 2 – 4 min). Then 100 µl/well of stop solution (Invitrogen, USA) was added and optical density was measured using the ELISA spectrophotometer Spark (Tecan, Switzerland) at 450 nm and at 620 nm as a reference wavelength. Data were analyzed by AccelPharm, Switzerland to derive pharmacokinetic parameters.

### Anti-tumor efficacy in metastatic Renca mouse model

The anti-metastatic efficacy of SOT101 was examined in a syngeneic Renca mouse model in female BALB/c mice under various treatment schedules and doses. Renca cells (CCL-2947™) were maintained *in vitro* with RPMI 1640 medium supplemented with 2 mM GlutaMAX I CTS, 100 U/ml penicillin + 100 mg/ml streptomycin, 1 mM Sodium pyruvate, 1% non-essential amino acids and with 10% FBS (all from Gibco, Thermo Fisher Scientific, USA) in an atmosphere of 5% CO_2_. Mice were obtained from the breeding colony at the Institute of microbiology of the Czech Academy of Sciences. The *in vivo* mouse experiments were conducted according to the institutional regulations compliant with the animal welfare policy and laws in the Czech Republic. Animal experimental plans were approved by the internal ethics committee. Renca cells in the exponential growth phase were harvested and quantitated by cell counter before tumor inoculation. Each mouse was inoculated intravenously into the tail vein with Renca tumor cells (1×10^5^) in 0.1 ml of PBS at day 0, 8 mice per group, 11-12 weeks of age. SOT101 was administered SC at the indicated doses daily for the stated consecutive or non-consecutive days (24 h apart between days) starting day 1. After 16 days the metastatic burden in lungs was determined. Lungs were gently taken out from mouse, placed into a microcentrifuge tube and the weight was determined. For flow cytometry analyses, mice were sacrificed on day 5 or 12 and splenic NK cells from 3-4 mice/group were analyzed as described above.

### Anti-tumor efficacy in human multiple myeloma xenograft tumor model *in vivo*

The anti-tumor efficacy of SOT101 and Daratumumab monotherapies and the combination was examined in a subcutaneous human multiple myeloma xenograft (RPMI8226) in female CB17 SCID mice under various treatment schedules. Daratumumab was injected IP at dose of 20 mg/kg on a single day. SOT101 was administered SC at dose of 1 mg/kg daily for four consecutive days (24 h apart). Studies were performed according to animal welfare regulations by CrownBio (Bejing, China). The RPMI 8226 tumor cells were maintained *in vitro* with RPMI 1640 medium supplemented with 10% FBS at 37°C in an atmosphere of 5% CO_2_. The cells in the exponential growth phase were harvested and quantitated by cell counter before tumor inoculation. Each mouse was inoculated subcutaneously in the right front flank region with RPMI 8226 tumor cells (1×10^7^) in 0.1 ml of PBS mixed with matrigel (1:1) for tumor development. All animals were randomly allocated to the different study groups (8 mice per group, 9-11 weeks of age). The mean tumor size at randomization was ~100 mm^3^. Randomization was performed based on “Matched distribution” randomization method (StudyDirector™ software, version 3.1.399.19). The date of randomization was denoted as day 1. Treatment was initiated on the randomization day 1 (early therapeutic treatment) or 5 days after randomization (late therapeutic treatment). After tumor cell inoculation, the animals were checked daily for morbidity and mortality. Tumor volumes were measured two times per week in two dimensions using a caliper, and the volume was expressed in mm^3^.

### Statistical analyses

Wilcoxon signed-rank test was applied for *in vitro* data analysis using GraphPad PRISM 6 (San Diego, California, USA). The results were considered statistically significant if * p< 0.0332, ** p< 0.0021, *** p< 0.0002, **** p< 0.0001. Two-way ANOVA was applied for mouse pharmacodynamic experiments. To compare tumor volumes of different groups, Bartlett’s test to check the assumption of homogeneity of variance across all groups was used. When the p-value of Bartlett’s test was ≥0.05, one-way ANOVA was applied. If the p-value of the one-way ANOVA was<0.05, *post hoc* Tukey’s HSD tests for all pairwise comparisons, and Dunnett’s tests for comparing each treatment group with the vehicle group was run. When the p-value of Bartlett’s test was<0.05, Kruskal-Wallis test was applied. If the p-value the Kruskal-Wallis test is<0.05, *post hoc* Conover’s non-parametric test for all pairwise comparisons or for comparing each treatment group with the vehicle group, both with single-step p-value adjustment, was run. In addition, pairwise comparisons without multiple testing correction and report nominal/uncorrected p-values directly from Welch’s t-test or Mann-Whitney U test was applied. Specifically, Bartlett’s test was used to check the assumption of homogeneity of variance for a pair of groups. When the p-value of Bartlett’s test was ≥0.05, Welch’s t-test was run, otherwise Mann-Whitney U test, to obtain nominal p-values was applied. All statistical analyses were done in R—a language and environment for statistical computing and graphics (version 3.3.1). All tests were two-sided unless otherwise specified, and the results were considered statistically significant if * p< 0.05, ** p< 0.01, *** p< 0.001 or **** p< 0.0001. The data are presented as mean ± SEM.

## Results

### SOT101 expands and activates human NK cell subtypes *in vitro*

**Figure 1:**
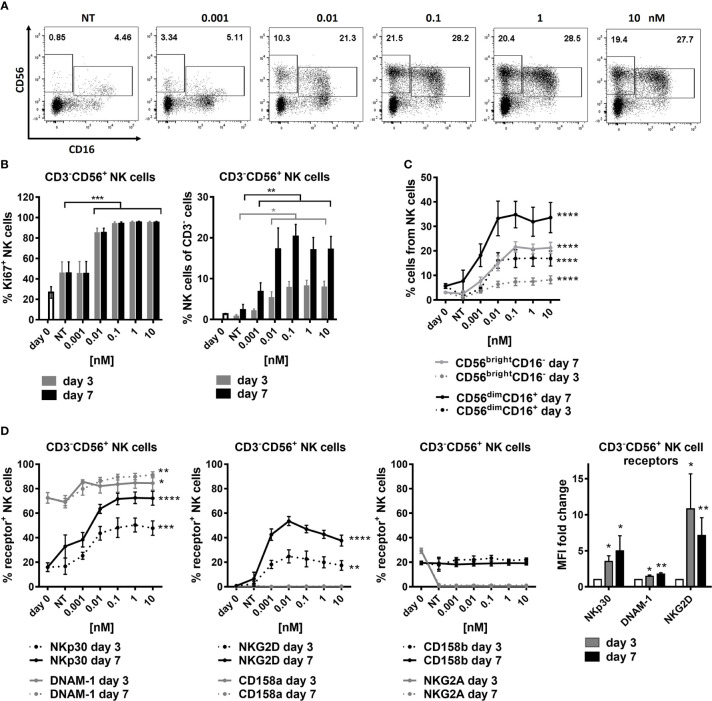
SOT101 expands and activates human NK cell subtypes *in vitro.* Human PBMCs were incubated with the increasing concentrations of SOT101 at 0.001 – 10 nM for 7 days (NT – non-treated). NK cells (CD3^-^CD56^+^) and their subpopulations (CD56^dim^CD16^+^ and CD56^bright^CD16^-^) were analyzed by flow cytometry at day 3 and 7. **(A)** representative dotplots from day 7 are shown **(B)** SOT101-stimulated proliferation and the relative expansion human NK cells and **(C)** their subpopulations CD56^dim^CD16^+^ and CD56^bright^CD16^-^ in a concentration-dependent manner already at day 3 *in vitro*. Statistical significance from SOT101 at 0.01 nM. NK cell proliferation was detected as a percentage of Ki67^+^ cells. **(D)** SOT101 increased the relative number of NK cells expressing the activating receptors DNAM-1, NKp30, and at low concentrations also NKG2D, and increased their expression on NK cells (fold change of MFI with SOT101 at 1 nM) already at day 3. Statistical significance from SOT101 at 0.01 nM for NKp30, DNAM-1 and from SOT101 at 0.001 nM for NKG2D. SOT101 did not change the expression levels of the inhibitory receptors NKG2A, CD158a and CD158b on NK cells. Data represent mean ± SEM from 4-8 healthy donors. The results were considered statistically significant if * p < 0.05, ** p < 0.01, *** p < 0.001 or **** p < 0.0001.

SOT101 had been shown to induce proliferation, expansion, and upregulation of activating receptors of mouse and human NK cells (9, 37). However, the studies did not differentiate between the two main human NK cell populations. Human PBMCs were incubated with the increasing concentrations of SOT101 at 0.001 – 10 nM for 7 days. NK cells (CD3^-^CD56^+^) and their subpopulations CD56^dim^CD16^+^ and CD56^bright^CD16^-^ were analyzed by flow cytometry at day 3 and day 7. As shown in **Figure 1** SOT101 induced a significant proliferation and an increase in relative numbers of NK cells in a concentration-dependent manner *in vitro* already after 3 days. EC50 of NK cell proliferation was 10 pM at day 7 (**Figures 1A, B**). Both NK cell subpopulations CD56^dim^CD16^+^ and CD56^bright^CD16^-^ were expanded by SOT101 to a similar extent, however a difference in their sensitivity to SOT101 concentrations was observed (**Figure 1C**). After 7 days the maximum relative expansion of CD56^dim^CD16^+^ NK cells was reached with SOT101 stimulation at 0.01 nM, one order of magnitude lower than the concentration needed for a maximum relative expansion of CD56^bright^CD16^-^ NK cells (0.1 nM). Representative dotplots of both NK cell subpopulations at day 7 are shown in **Figure 1A**. Further, we analyzed a concentration-dependent expression of several activating and inhibitory receptors on NK cells after 3 and 7 days of SOT101 stimulation *in vitro* (**Figure 1D**). SOT101 treatment did not alter the expression of inhibitory receptors NKG2A, CD158a and CD158b on NK cells at day 3 or 7 of an *in vitro* stimulation. On the other hand, SOT101 expanded the relative numbers of human NK cells expressing activation receptors DNAM-1, NKG2D and NKp30 and increased their expression as shown by fold change MFI (with SOT101 at 0.1 nM) on NK cells already after 3 days *in vitro*. Interestingly, the maximum increase in the relative number of NKG2D-expressing NK cells was observed upon SOT101 stimulation at 0.01 nM and decreased with higher SOT101 concentrations in contrast to the expression of DNAM-1 and NKp30. Altogether, these data show that SOT101 stimulates both human NK cell subpopulations and increases the expression of receptors mediating NK cell cytotoxicity.

### SOT101 induces cytotoxic and tumor cell-killing activity of human NK cell subtypes *in vitro*

**Figure 2:**
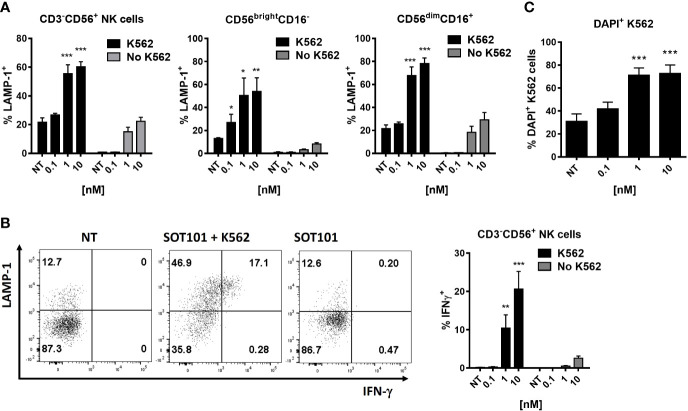
SOT101 induces cytotoxic and tumor cell-killing activity of human NK cell subtypes *in vitro*.PBMCs were pre-stimulated with 0, 0.1, 1 and 10 nM SOT101 for 3 days, then added to the target tumor cells DiD^+^K562 at E:T ratio 1:5. The cytotoxic activity was determined by **(A)** LAMP-1 degranulation on NK cells and their subpopulations and **(B)** by an intracellular IFN-γ staining after 4h. **(C)** The percentage of killed DiD^+^DAPI^+^ K562 cells was determined after 6 h by flow cytometry. Representative dotplots of LAMP-1 and IFN-γ staining in NK cells are shown in **(B)** (NT – non-treated NK cells with K562; SOT101 + K562 – NK cells pre-stimulated with SOT101 at 1 nM with K562; SOT101 – NK cells pre-stimulated with SOT101 at 1 nM without K562). Data represent mean ± SEM from 4 healthy donors. The results were considered statistically significant if * p < 0.05, ** p < 0.01 or *** p < 0.001.

Next, we analyzed if SOT101-stimulated NK cells and specifically their subpopulations exert cytotoxic activity towards cancer cells *in vitro*. Human PBMC were pre-stimulated with SOT101 at 0.1, 1 and 10 nM for 3 days *in vitro* and then added to DiD-stained K562 tumor cells at E:T ratio 1:5. NK cell cytotoxicity was determined by staining a marker of cytotoxic degranulation LAMP-1 and by the intracellular IFN-γ staining after 4 h, and the percentage of killed K562 tumor cells (DiD^+^DAPI^+^) was determined after 6 h by flow cytometry. As shown in **Figure 2** NK cells and both their subpopulations CD56^bright^CD16^-^ and CD56^dim^CD16^+^ exhibited a significant cytotoxic activity towards K562 tumor cells at 1 nM as detected by LAMP-1 degranulation (**Figure 2A**) and IFN-γ production (**Figure 2B**). Similarly, NK cells and both their subpopulations CD56^bright^CD16^-^ and CD56^dim^CD16^+^ significantly increased the percentage of killed K562 tumor cells (**Figure 2C**). This suggests that both subtypes of NK cells are capable of a cytotoxic function and killing of tumor cells when activated with SOT101.

### SOT101 enhances activity of therapeutic antibodies in human PBMC and NK cell killing assays *in vitro*

**Figure 3:**
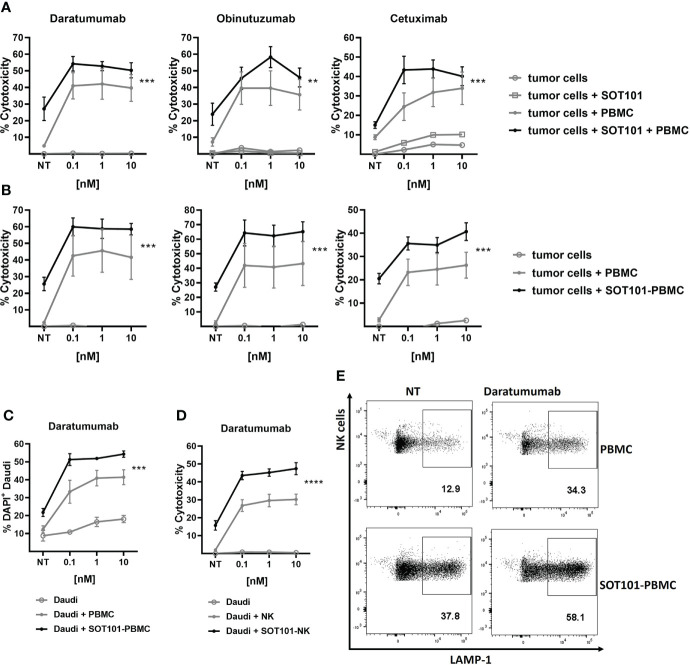
SOT101 enhances the tumor cell killing activity of therapeutic antibodies *in vitro* **(A)** Concomitant treatment: PBMCs were added to the target tumor cells together with SOT101 at 1 nM and the therapeutic antibodies at 0.1, 1 and 10 nM and incubated for 20 h E:T ratio was 25:1 for Daudi (Daratumumab, Obinutuzumab) and 150:1 for SCC25 (Cetuximab) cells, respectively. **(B)** Sequential treatment: PBMCs were first pre-stimulated with 0.1 nM SOT101 for 3 days (SOT101-PBMC) and then added to the target tumor cells together with therapeutic antibodies at 0.1, 1 and 10 nM and incubated for 24 h E:T ratio was 15:1 for Daudi and 100:1 for SCC25 cells, respectively. The percentage of cytotoxicity was determined by a LDH release assay. **(C, E)** PBMCs were pretreated with SOT101 (0.1 nM, 3 days) and then incubated with Daratumumab and Daudi cells (E:T 1:1) for 4 h **(C)** The percentage of dead DiD^+^DAPI^+^ tumor cells was determined by flow cytometry. **(E)** Representative dotplots for NK cell degranulation as determined by CD3^-^CD56^+^LAMP-1^+^ staining Daratumumab was used at 0.1 nM here. **(D)** Isolated NK cells were pretreated with SOT101 (0.1 nM, 2 days) and then incubated with Daratumumab (0.1, 1 and 10 nM) and Daudi tumor cells (E:T 10:1) for 4 h. The percentage of cytotoxicity was determined by a LDH release assay. Data represent mean ± SEM from 3-6 healthy donors. The results were considered statistically significant if ** p < 0.0021, *** p < 0.0002 or **** p < 0.0001.

We examined if SOT101-stimulated PBMC can synergize with several clinically approved monoclonal antibodies *in vitro*. Human PBMC and tumor cells were incubated either concomitantly (**Figure 3A**) or sequentially (**Figure 3B**). In a concomitant setting SOT101 at 1 nM and the marketed therapeutic anti-CD38 antibody Daratumumab, the anti-CD20 antibody Obinutuzumab or the anti-EGFR antibody Cetuximab at 0.1, 1 and 10 nM were incubated with tumor cells for 20 h. In a sequential setting, PBMC were pre-stimulated with SOT101 at 0.1 nM for 3 days and then added to tumor cells together with the respective monoclonal antibody at 0.1, 1 and 10 nM for 24 h. In both settings, SOT101-activated PBMC significantly increased the therapeutic effect of monoclonal antibodies at concentration as low as 0.1 nM to kill tumor cells in comparison to controls as determined by LDH release assay. Direct flow cytometric detection of killed tumor cells upon incubation with Daratumumab and SOT101-pretreated PBMC after 4 h incubation is shown in **Figure 3C**. In line with this observation, a stronger degranulation of human NK cells was detected by LAMP-1 staining when compared to single treatments (**Figure 3E**). To show specifically that SOT101-stimulated NK cells account for the potentiation of the killing activity of therapeutic antibodies, isolated human NK cells were preincubated with SOT101 at 0.1 nM for 2 days and then added with Daratumumab at 0.1, 1 and 10 nM to Daudi tumor cells for 4 h. As shown in **Figure 3D** SOT101-prestimulated human NK cells significantly increased tumor cell killing when combined with various concentrations of Daratumumab over the monotherapies alone. These data confirm that SOT101-stimulated NK cells potentiate tumor cell killing mediated by the therapeutic antibodies.

### SOT101 activates NK cells and induces anti-tumor efficacy in dependance on dose, administration route and schedule *in vivo*

**Figure 4:**
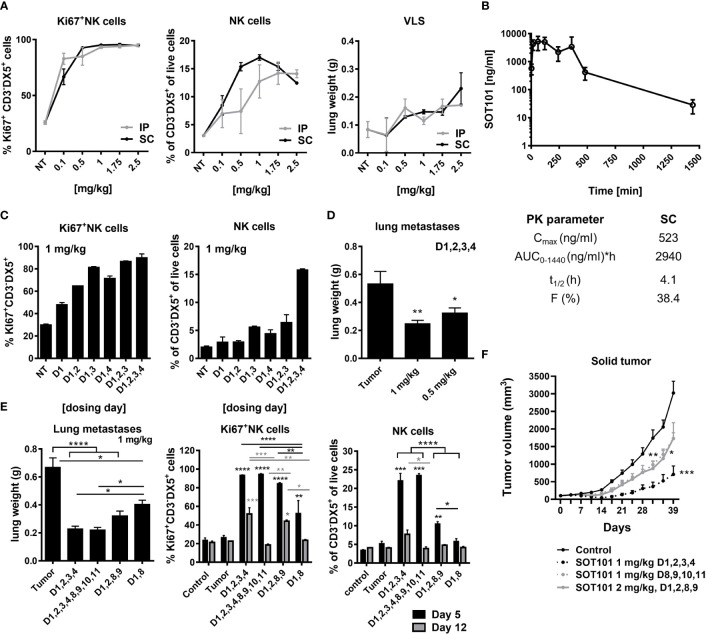
SOT101 activates NK cells and induces anti-tumor efficacy in dependance on dose, administration route and schedule *in vivo*. **(A)** SOT101 was administered IP or SC once daily at the indicated doses into C57BL/6 mice at day 1,2,3,4 in 2 mice/group. NK cell proliferation and the relative expansion was analyzed from splenocytes by flow cytometry at day 5. Lung weight was determined at day 5 to detect vascular leakage syndrome (VLS). **(B)** SOT101 was administered SC at 2.5 mg/kg in 2 mice per time point. Serum was collected at 2 min, 15 min, 1 h, 2 h, 4 h, 6 h, 8 h, 24 h and 32 h after dose administration and analyzed by ELISA. Pharmacokinetic parameters were calculated. **(C)** SOT101 was administered SC at 1 mg/kg once daily at the indicated day. NK cell proliferation and the relative expansion was determined by flow cytometry at day 5. **(D)** SOT101 was administered SC at 0.5 or 1 mg/kg at day 1,2,3,4 in the Renca mouse tumor model. Lung metastases were evaluated as the lung weight at day 16. **(E)** Dosing schedule dependency of SOT101 at 1 mg/kg SC in Renca mouse model for prevention of lung metastases. NK cell activity from spleen was evaluated at day 5 and 12. **(F)** Dose and schedule dependency of SOT101 administered SC in solid multiple myeloma xenograft tumor model. Data represent mean ± SEM from 2-4 mice/group for PD and PK, and 8 mice/group for the anti-tumor efficacy experiments *in vivo*. The results were considered statistically significant if * p < 0.05, ** p < 0.01, *** p < 0.001 or **** p < 0.0001.

Pharmacodynamic activity and toxicity of SOT101 at various doses, route of administration and schedules were investigated in healthy C57BL/6 mouse and in metastatic mouse Renca and solid human multiple myeloma RPMI 8226 xenograft tumor model *in vivo* to establish an optimal therapeutic regiment of SOT101 for combination with Daratumumab. Previously it had been shown that SOT101 induced accumulation, proliferation, maturation, and cytotoxic function of NK cells in tumor-free mice at day 5 upon intraperitoneal (IP) administration of 2 µg/mouse (~0.1 mg/kg) at day 1,4 (37). Here, SOT101 was administered IP or SC at various doses (0.1 – 2.5 mg/kg) as a once daily injection for four consecutive days (day 1, 2, 3, 4 – 24 h apart between doses). The NK cell proliferation, relative expansion and potential SOT101 toxicity as determined by lung wet weight as a marker for vascular leakage syndrome (VLS) onset were compared at day 5 (**Figure 4A**). A high dose-dependent NK cell proliferation in spleen was detected upon SOT101 IP and SC administration, however with a higher NK cell expansion upon SOT101 SC administration peaking at dose 1 mg/kg which was selected for further experiments. Mice did not exhibit any clinical signs of toxicity; however, the lung wet weight (VLS) was markedly higher at the highest SOT101 dose of 2.5 mg/kg (**Figure 4A**). Pharmacokinetics of SOT101 was investigated upon a single SC administration of 2.5 mg/kg and the parameters such as C_max_, half-life (t_1/2_), AUC and bioavailability (F) were determined (**Figure 4B**). The half-life of SOT101 was determined to be 4.1 h, C_max_ was 523 ng/ml, AUC 2940 ng/ml*h and bioavailability 38.4%. Next, various dosing days/schedules of SOT101 at the selected dose of 1 mg/kg administered SC were compared at day 5. As shown in **Figure 4C** increased NK cell proliferation was detected already after one dose of SOT101 and rapidly increased in other dosing schedules. However, the highest expansion of NK cells was detected when SOT101 was administered at day 1,2,3,4 in comparison to a lesser day-dense schedules. Additional dosing days were not tested due to the only minor further increase in Ki67^+^ NK cells between the dosing schedules (D1,2,3 and D1,2,3,4). The SOT101 dose effect on anti-tumor efficacy was evaluated in the metastatic Renca mouse model as lung weight after 16 days (**Figure 4D**). SOT101 at 0.5 mg/kg dosed day 1,2,3,4 significantly decreased the development of lung metastasis similarly to a previously described effect of SOT101 in B16F10 melanoma metastatic model (11) which suggests a high potency of SOT101 also in the Renca model. Schedule-dependent anti-metastatic efficacy was evaluated upon SC administration of SOT101 at the dose of 1 mg/kg. As shown in **Figure 4E** SOT101 administered at all schedules significantly reduced lung metastatic burden. However, only SOT101 treatment at day 1,2,3,4 induced the optimal and persistent NK cell activity up to day 12. Interestingly, additional dosing in the 2^nd^ week (D1,2,3,4,8,9,10,11), induced significantly lower NK cell proliferation and relative expansion at day 12 in comparison to SOT101 dosing 1^st^ week only (D1,2,3,4) despite similarly effective anti-metastatic activity. Further, SOT101 doses of 1 mg/kg or 2 mg/kg of SOT101 at selected schedules were tested in solid human multiple myeloma RPMI 8226 xenograft tumor model in CB17 SCID mice. As shown in **Figure 4F** SOT101 dosed at 1 mg/kg day 1,2,3,4 induced the strongest anti-tumor efficacy in this model. The treatment started at randomization day 1 with tumor volumes of ~100 mm^3^. No clinical signs of toxicity such as weight loss were observed in experiments in **Figures 4E, F**. Overall, it can be concluded that the identified dosing schedule day 1,2,3,4 of SOT101 administration SC in mice induces optimal NK cell activation and low toxicity at a tolerable number of administrations leading to a superior anti-tumor activity in comparison to other tested schedules.

### SOT101 increases therapeutic activity of Daratumumab in a solid human multiple myeloma xenograft model *in vivo* in dependance on the combination schedule

**Figure 5:**
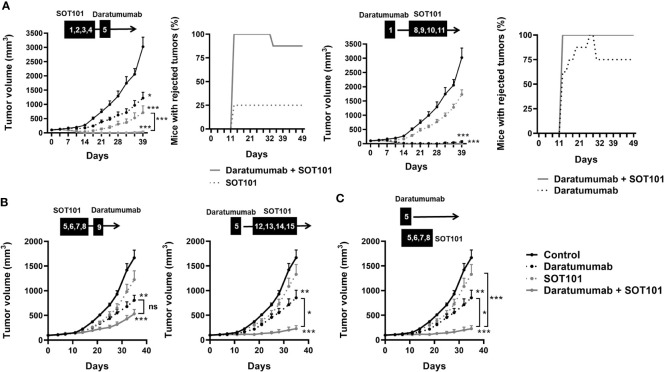
SOT101 potentiates the therapeutic activity of Daratumumab in a solid human multiple myeloma xenograft model *in vivo* in dependence on the combination schedule. SOT101 was administered SC at 1 mg/kg and Daratumumab was administered IP at 20 mg/kg at the indicated timepoints **(A)** sequential drug schedules in the early therapeutic setting where treatment started at randomization day 1 (~ 100 mm^3^). **(B)** sequential drug schedules in the late therapeutic setting where the treatment started 4 days post randomization (day 5) **(C)**. Concomitant drug schedule in the late therapeutic setting. Data are presented as mean ± SEM from 8 mice and show a representative study of n = 2. The results were considered statistically significant if * p < 0.05, ** p < 0.01 or *** p < 0.001; ns, not significant.

The therapeutic benefit of SOT101 in combination with Daratumumab was investigated in a human multiple myeloma xenograft tumor model *in vivo* testing several combination treatments schedules. The established tumors were treated with Daratumumab at 20 mg/kg and SOT101 at 1 mg/kg based on the tumor advancement at the randomization day 1 (~100 mm^3^) as early therapeutic setting or at day 5 (4 days post-randomization) as late therapeutic setting (**Figure 5**). In the early therapeutic setting, SOT101 monotherapy significantly decreased the tumor volume only when administered at day 1, 2, 3, 4 but not later at day 8,9,10,11. In contrast Daratumumab monotherapy significantly reduced tumor growth when administered at day 1 or day 5. The additive effect of the combination of both drugs could be seen only at the level of tumor regression where both tested combination schedules led to a higher number of cured mice in comparison to monotherapies (**Figure 5A**). In the late therapeutic setting SOT101 monotherapy did not show any anti-tumor efficacy, in contrast to Daratumumab monotherapy. This would be in line with a lack of efficacy of SOT101 monotherapy observed at days 8,9,10,11 in **Figure 5A** which could be in this sense also considered as late therapeutic treatment. The combination of both drugs in the late therapeutic setting, at the sequential schedule Daratumumab (day 5) followed by SOT101 treatment (day 12,13,14,15), showed a significant tumor volume reduction (**Figure 5B**), however no tumor regressions were observed (data not shown). Further, in this late therapeutic setting a concomitant administration of both drugs was investigated (**Figure 5C**). Here the combination of Daratumumab (day 5) and SOT101 (day 5,6,7,8) significantly reduced tumor growth in comparison to respective monotherapies. The data suggest that SOT101 monotherapy can be effective in early tumor treatment, however, might not be effective in advanced developed tumors. SOT101 can significantly enhance the therapeutic effect of Daratumumab, specifically in schedule where Daratumumab is followed by SOT101 administration or as a concomitant administration of both drugs.

## Discussion

SOT101 represents a promising IL-15-based immunotherapeutic drug for the treatment of cancer. In this study we have shown that SOT101 expanded and activated both subpopulations of human NK cells CD56^bright^CD16^-^ and CD56^dim^CD16^+^ and their cytotoxic activity towards tumor cells *in vitro*. SOT101 further potentiated the activity of several clinically approved monoclonal antibodies to kill tumor cells by activating NK cells *in vitro*. A favorable SOT101 dosing schedule to effectively activate NK cells for treatment of solid tumors in mouse models together with a subcutaneous (SC) route of SOT101 administration has been investigated. The efficacy of SOT101 monotherapy using a selected schedule and dose was confirmed in the treatment of a solid multiple myeloma xenograft tumor model *in vivo*. Most importantly, in this model SOT101 and Daratumumab treatment exhibited a significant additive anti-tumor efficacy upon a sequential and concomitant administration of Daratumumab and SOT101.

Human NK cells are major effectors of innate immunity and are also pivotal players in cancer immunotherapy. In humans NK cell subpopulations have been defined based on the levels of CD56 expression. CD56^dim^ NK cells are considered the cytotoxic NK cell subset, whereas CD56^bright^ NK cells were originally believed to be a predominantly cytokine-producing immunoregulatory subset without a significant cytotoxic function. The numbers of CD56^bright^ NK cells are estimated to be substantially higher than those of CD56^dim^ NK cells (25). Higher relative numbers of CD56^bright^ NK cells with cytotoxic function were expanded by SOT101 compared to CD56^dim^ NK cells. This is in line with similar *in vitro* observations with IL-15 or another IL-15-based agonist (29). Moreover, in patients with metastatic malignancies, IL-15 administration *via* continuous intravenous infusion at 20 μg/kg/day led to a 38-fold increase in number of circulating NK cells, and specifically the number of CD56^bright^ NK cells increased 358-fold (28). The sensitivity of CD56^bright^ NK cells to SOT101-induced proliferation seems to be higher than for CD56^dim^ NK cells which might point to a possible higher CD122 receptor expression on these cells, hence to a higher responsiveness (25). The IL-15-stimulated effector mechanisms of NK cell cytotoxicity were proposed to include improved target cell conjugation, cytotoxic protein expression, cytokine production and LFA-1, CD2-, and NKG2D-dependent activation of NK cells (29). In our study we observed an increase in cytotoxic receptors including NKp30, DNAM-1 and specifically with low concentrations of SOT101 also the NKG2D receptor. We further detected an increased IFN-γ production and NK cell degranulation (LAMP-1^+^) upon SOT101 treatment.

SOT101 induced an increase of the relative numbers of NK cells and the augmentation of the activation state of NK cell was also associated with an increase in the tumor cell-killing activity of three monoclonal ADCC-inducing antibodies Cetuximab, Daratumumab and Obinutuzumab *in vitro*. ADCC represents a pivotal mechanism of anti-tumor action of these monoclonal therapeutic antibodies (31, 32). IL-15 has been shown to increase Cetuximab-mediated cellular toxicity against triple-negative breast cancer cell lines expressing EGFR (35) and Rituximab-mediated cellular toxicity against B cell lymphoma cell expressing CD20 (34). Similarly, IL-15-based agonists enhanced the NK cell response to Cetuximab-treated squamous cell carcinoma of head and neck (38) and colon cancer (31) and to Rituximab-treated B cell lymphoma cell lines (36). However, the respective contribution of the enhanced ADCC mechanism *via* CD16 receptors versus the increased direct toxicity of IL-15-stimulated NK cells has not been determined by these studies. The increased expression of CD16 receptor on NK cells was not detected in our study (data not shown) which is in line with a previous observation with SOT101 (37). The crosslinking of CD16 receptors together with engagement of the IL-15Rβγ has been shown to increase a cytotoxicity of NK cells after the incubation with an Fc-part lacking trispecific agent (39) or after stimulation with a murine IL-15/IL-15Rα-Fc complex having a human IgG1 Fc isotype (40). Here the dependence on the Fc receptor binding mechanism was confirmed in FcγR−/− mice. If such mechanism applies for free antibody and SOT101 combinations in not clear but it is plausible that the tight crosslinking could be achieved only by such Fc fusion molecules.

The potentiation of anti-tumor efficacy of monoclonal therapeutic antibodies by IL-15 or IL-15-based agonists has also been observed in mouse models *in vivo* (31, 36, 38). Administration of Cetuximab and IL-15 superagonist N-803 (formerly ALT-803) in mice harboring Cal27 squamous cell carcinoma of head and neck tumors significantly decreased tumor volume when compared to controls and single-agents treatment alone (38). Similarly, coinjection of Rituximab and N-803 resulted in an increased survival of the experimental animals compared to controls or monotherapies (36). Most of these experiments were conducted in partially immunocompetent SCID or nude mice. However, an increased anti-tumor efficacy was observed upon treatment with Rituximab and N-803 in immunodeficient mice reconstituted with human NK cells (36) or with Cetuximab and Sushi IL-15-Apo protein in human PBMC reconstituted mice bearing HT-29 colon cancer xenografts (31). To investigate on the additive effect of SOT101 and ADCC-inducing monoclonal antibodies treatment, the dose and schedule-dependent effect of SOT101 on NK cells was determined in mice *in vivo*. SOT101 administered SC induced higher relative expansion of mouse NK cell when compared to IP administration. The optimal NK cell expansion was observed only when SOT101 was administered once daily on four consecutive days at selected dose of 1 mg/kg. Increased lung weight was observed with the highest dose tested at 2.5 mg/kg; however, no clinical signs of toxicity were observed. High toxicity was observed upon IP administration of a mouse IL-15/IL-15Rα-Fc human IgG1 complex dosed at 2µg/mouse (~0.1 mg/kg) once daily on four consecutive days (41). This was most likely caused by a substantially higher stimulation of the target cells due to a longer half-life of this ~ 90-100 kDa-sized molecule compared to 23 kDa of SOT101. The half-life of SOT101 administered SC as a single dose was 4.1 h which is slightly higher than 3 h after IP administration of SOT101 at similar dose tested previously (11). The relatively short half-life of SOT101 seems to be rather beneficial on NK cells as a repetitive short-term IL-15 stimulation was shown to activate NK cells with better fitness and effector functions in comparison to a long persistent IL-15 activation, which induced NK cell exhaustion (42, 43). SOT101 at 1 mg/kg administered once daily on four consecutive days within the 1^st^ week induced a strong anti-metastatic efficacy in Renca mouse model with a high NK cell proliferation and expansion which was still observed at day 12. NK cell proliferation and the relative expansion was however decreased at day 12 when SOT101 was administered SC once daily on four consecutive days during the 1^st^ and 2^nd^ week. This suggests that too frequent SOT101 treatment at this dose of 1 mg/kg can lead to lower NK cell responsiveness possibly caused by NK cell exhaustion. However, this remains to be further investigated as we observed a comparable strong anti-metastatic efficacy of SOT101 for the two-week treatment similarly to a one-week treatment only. NK cell hyporesponsivness upon just two administration of IL-15 (day 1 and 3) was shown only for dose of 10 µg/mouse (~0.5 mg/kg) (44). Given the IL-15 half-life of ~30 min in mice (45) the pharmacodynamic effect would be far lower than the effect caused by SOT101 at 1 mg/kg with half-life of 4.1 h after SC administration. It is not clear if the observed hyporesponsiveness concerns only a low IL-15-mediated NK cell stimulation and may not apply to a IL-15 stimulation above a certain dose threshold as it has been documented for endotoxin (LPS) (46). SOT101 inducing a strong anti-metastatic efficacy in the syngeneic Renca mouse model confirms also previous findings with SOT101 on metastases decrease in B16F10, 4T1 and HCT-116 mouse models (11, 37). Moreover, in the B16F10 mouse model it was shown that the SOT101-induced anti-metastatic efficacy was dependent on NK cells as confirmed by NK cell depletion experiments (11). The control of metastatic dissemination in the Renca mouse model was also shown to be dependent on the activity of NK cells (47). Although SOT101 administered IP at dose of 2 µg/mouse (~ 0.1 mg/kg) as frequent as three or four times per week over the course of 3 to 4 weeks effectively reduced metastases in orthotopic HCT-116 or 4T1 mouse tumor models, it displayed little or no effect on the primary solid tumor mass (11, 37). Using a solid human multiple myeloma xenograft tumor model in partially immunocompetent SCID CB17 mice, we identified that SOT101 at 1 mg/kg administered SC once daily at four consecutive days can also significantly decrease the growth of established tumors. This created a base for a subsequent investigation on the anti-tumor efficacy of SOT101 and Daratumumab combination in this model. A significant anti-tumor efficacy was observed upon treatment with SOT101 and Daratumumab. Based on the previous findings it is likely that a similar potentiation of the anti-tumor efficacy could be expected also for other ADCC-inducing antibodies when combined with SOT101 *in vivo*. More importantly, several schedules of combination treatment of Daratumumab and SOT101 were investigated and two were identified to significantly decrease the tumor volume in the late therapeutic setting of established tumors: a sequential addition of Daratumumab followed by SOT101 and a concomitant administration of both drugs. This suggests that a certain schedule flexibility in the administration of both drugs is feasible which may be important in the clinics where drugs are administered in cycles. In multiple myeloma, Daratumumab is administered at 16 mg/kg weekly for the initial 8 weeks and then the administration is decreased to every two, and later four weeks cycles. This would be fully compatible with the clinical schedule of SOT101 employed in the ongoing Phase II clinical trial in combination with pembrolizumab in patients with selected advanced solid tumors (NCT05256381). Since the SOT101 doses in mice needed to be higher due to a lower homology of the IL-15Rβγ compared to the human receptors, the SOT101 doses and schedule were adapted and optimized in a dose escalation Phase I clinical trial (NCT04234113). Our preclinical data shown in this study would support such trial design also with other therapeutic monoclonal antibodies. Given the fact that ADCC is also the major mechanism of other therapeutic antibodies such as Cetuximab, Obinutuzumab, Rituximab etc. similar schedule of their combination with SOT101 could be proposed. Whereas the combination therapy was very effective inducing tumor regressions in comparison to respective monotherapies, in the early therapeutic treatment of the established tumors no dependence on the sequencing of both drugs was observed. 95% of animals treated with SOT101 and Daratumumab showed tumor regression, which supports even higher schedule flexibility when the tumor burden would be low in patients.

In the multiple myeloma xenograft tumor model in SCID CB17 mice the adaptive parts of immunity such as T cells are missing to support anti-tumor efficacy upon SOT101 treatment as SOT101 also efficiently stimulates effector and memory cytotoxic CD8^+^ T cells *in vivo* (12). NK cells, macrophages and granulocytes are the only immune cell populations capable of mediating an anti-tumor effect upon Daratumumab and SOT101 therapy in this model. Despite differences between mouse and human antibody isotypes, human IgG1 is the most potent human IgG isotype in tumor models in mice (48). ADCC in mice *via* human IgG1 antibodies is mediated by FcγRI and FcγRIV receptors on NK cells and macrophages (49). Interestingly, both NK cells and macrophages were shown to be necessary, but not alone sufficient, for an optimal ADCC upon the treatment with IL-15 and human IgG1 therapeutic antibodies in mouse tumor models *in vivo* (50). Although the *in vivo* anti-tumor efficacy mechanisms of SOT101 and Daratumumab have not been characterized in this study in detail, it is hypothesized that activated NK cell-mediated ADCC, and macrophage-dependent cellular phagocytosis (ADCP) underlie the observed anti-tumor effects together with SOT101-induced activation of NK cell cytotoxic functions, IFN-γ production and the expression of activating receptors. In addition, SOT101 was shown to decrease suppressive granulocytes in mouse tumors *in vivo* (37), which may also potentially contribute to the anti-tumor efficacy observed in our selected model.

IL-15 was found to significantly augment the magnitude and duration of the anti-tumor response in the EL4-hCD20 syngeneic model treated with Rituximab and the ALT MET-1 xenograft model treated with Alemtuzumab (50). Interestingly, most of the therapeutic efficacy in the EL4-hCD20 model and all the therapeutic efficacy in the ATL MET-1 xenograft model was lost when models were established, and the treatment was conducted, in FcγR−/− mice. This supported the notion that the anti-tumor efficacy mediated by the combination of IL-15 and monoclonal antibodies was predominantly dependent on the ADCC. Here the NK cell interaction and the presence of macrophages was shown to be indispensable for the anti-tumor efficacy, as the clodronate treatment leading to the elimination of macrophages *in vivo* abrogated the IL-15 induced FcγRIV and NKG2D expression on NK cells (50). Therefore, both NK cells and macrophages were critical for the optimal therapeutic responses mediated by Rituximab in a combination regimen with human IL-15 (50). It is likely that a similar mechanism as described for human IL-15 and rituximab is applicable also to SOT101 and Daratumumab treatment in our study. On the other hand, in syngeneic fully immunocompetent mice, there would be other mechanisms additionally employed by SOT101 which would contribute to ADCC of the therapeutic antibodies and anti-tumor efficacy such as activation of NKT cell, γδ T cells and stimulation of cytotoxic CD8^+^ T cells (12). SOT101 was shown to induce anti-tumor efficacy in several syngeneic mouse models such as CT26 or MC38 where the CD8^+^ T cell activation is known to play a major role in the control of tumor growth (12).

In summary, the present study supports clinical investigations that involves SOT101 administration in association with tumor-directed monoclonal antibodies exerting ADCC in the treatment of patients with advanced cancer malignancy and identifies favorable schedules for the clinical trial designs.

## Data availability statement

The datasets presented in this article are not readily available because the data are proprietary of SOTIO Biotech AG. Requests to access the datasets should be directed to adkins@sotio.com.

## Ethics statement

The animal studies were reviewed and approved CrownBio and the Animal Welfare Committee of the Institute of Microbiology of the Czech Academy of Sciences, in accordance with the Guidelines for the Care and Use of Laboratory Animals, the Act of Czech National Assembly, the Collection of Laws no. 246/1992. Permissions no. 110/2016 were issued by the Animal Welfare Committee of the Institute of Microbiology of the Czech Academy of Sciences in Prague.

## Author contributions

IA coordinated the studies, analyzed the experimental data, and wrote the paper. JT performed the experiments *in vivo*. ZA, NP, KA, KS, EN, and MS conducted the *in vitro* experiments and analyzed the data. ZA, IA, GM, UM, RS, DB, MK, and JT contributed to the experimental designs, discussion of the results and edited the paper. All authors contributed to the article and approved the submitted version.

## Funding

This work was supported by Cytune Pharma and SOTIO Biotech AG.

## Conflict of interest

Authors ZA, NP, KA, KS, EN, UM, RS, and IA were employed by SOTIO Biotech a.s. Authors GM and DB were employed by Cytune Pharma.

The remaining authors declare that the research was conducted in the absence of any commercial or financial relationships that could be construed as a potential conflict of interest.

## Publisher’s note

All claims expressed in this article are solely those of the authors and do not necessarily represent those of their affiliated organizations, or those of the publisher, the editors and the reviewers. Any product that may be evaluated in this article, or claim that may be made by its manufacturer, is not guaranteed or endorsed by the publisher.
